# Recently Reported Biological Activities and Action Targets of Pt(II)- and Cu(II)-Based Complexes

**DOI:** 10.3390/molecules29051066

**Published:** 2024-02-29

**Authors:** Cristhian Eduardo Maciel-Flores, Juan Antonio Lozano-Alvarez, Egla Yareth Bivián-Castro

**Affiliations:** 1 Centro Universitario de los Lagos, Universidad de Guadalajara, Av. Enrique Díaz de León 1144, Col. Paseos de la Montaña, Lagos de Moreno 47460, Jalisco, Mexico; cristhian.maciel3112@academicos.udg.mx; 2 Departamento de Ingeniería Bioquímica, Universidad Autónoma de Aguascalientes, Av. Universidad 940 Cd. Universitaria, Aguascalientes 20131, Aguascalientes, Mexico; lozanoalvarez@yahoo.com

**Keywords:** platinum, copper, complexes, coordination compounds, bioinorganic, biological activity, antineoplastic, cancer

## Abstract

Most diseases that affect human beings across the world are now treated with drugs of organic origin. However, some of these are associated with side effects, toxicity, and resistance phenomena. For the treatment of many illnesses, the development of new molecules with pharmacological potential is now an urgent matter. The biological activities of metal complexes have been reported to have antitumor, antimicrobial, anti-inflammatory, anti-infective and antiparasitic effects, amongst others. Metal complexes are effective because they possess unique properties. For example, the complex entity possesses the effective biological activity, then the formation of coordination bonds between the metal ions and ligands is controlled, metal ions provide it with extraordinary mechanisms of action because of characteristics such as d-orbitals, oxidation states, and specific orientations; metal complexes also exhibit good stability and good physicochemical properties such as water solubility. Platinum is a transition metal widely used in the design of drugs with antineoplastic activities; however, platinum is associated with side effects which have made it necessary to search for, and design, novel complexes based on other metals. Copper is a biometal which is found in living systems; it is now used in the design of metal complexes with biological activities that have demonstrated antitumoral, antimicrobial and anti-inflammatory effects, amongst others. In this review, we consider the open horizons of Cu(II)- and Pt(II)-based complexes, new trends in their design, their synthesis, their biological activities and their targets of action.

## 1. Introduction

Most drugs on the market today are of organic origin, so the design of novel drugs with metal ions in their structure may be a valuable area of research. Such ions provide mechanisms of action that drugs of organic origin do not present [1]. This is because they exhibit wide spectra of coordination numbers and geometries that give rise to versatile molecular structures with modulated reactivity which is mediated by the specific selection between different transition metals and ligands [1,2,3]. Furthermore, metal ions can provide metallodrugs with certain kinetic and catalytic properties due to the exceptional property of certain metals to alter their own electronic structure, as well as the electronic structures of their ligands [2,4].

Compounds with metal ions have been widely used as antitumor agents; among these, platinum(II)-based drugs stand out. These originated with the discovery of the biological activity of cis-diaminodichloridoplatinum(II) (cisplatin) by Rosenberg in 1965. Cisplatin, also known as Platinol^®^, was approved by the U.S. Food and Drug Administration (FDA) for clinical use in 1978; it is currently considered by the World Health Organization (WHO) as an essential drug in the treatment of different types of neoplasia [5,6].

**Figure 1:**
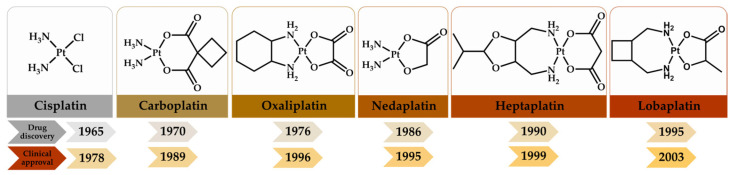
Structures of different platinum(II)-based antineoplastic drugs, the years in which they were discovered and the years in which they were approved.

The widespread use of cisplatin earned it the nickname “penicillin of cancer”. It also generated the design of new derivatives and similar antitumor agents [5]; these include compounds such as carboplatin and oxaliplatin, also considered essential medicines by the WHO [6], as well as nedaplatin, heptaplatin and lobaplatin. Figure 1 shows the discovery and clinical approval dates for six platinum(II)-based antineoplastic drugs. It can be seen that the periods required for clinical approval ranged from 8 to 20 years. In 1978, cisplatin was approved for use in clinical practice. Twenty-five years later, in 2003, lobaplatin was approved in China. In Mexico, the Federal Commission for the Protection against Health Risks (COFEPRIS, https://www.gob.mx/cofepris accessed on 18 February 2024) includes on its list of authorized medicines only three platinum(II)-based drugs: cisplatin, carboplatin and oxaliplatin [1,5,7].

It is estimated that around 50% of patients diagnosed with some type of cancerous tumor are now treated with platinum(II)-based drugs [8], the most commonly used of which are cisplatin and carboplatin. These are used in the treatment of various human cancers, including ovarian, testicular, bladder, colorectal, lung, head, neck and pancreatic cancers [5,9]. In addition, their effectiveness against some cancer cells, including carcinomas, germ-cell tumors, lymphomas and sarcomas, has also been reported [5].

However, it is well known that platinum(II)-based complexes provoke a wide range of side effects such as gastrointestinal, hematological, nephrological and neurological toxicity, as well as drug resistance [1,10]. To overcome these limitations, different strategies have been developed, such as the use of different classes of ligands and metal ions that could provide desirable characteristics to complexes. Such characteristics include increased solubility, specificity, effectiveness and stability. Ligands such as phosphines [11,12,13,14,15,16], dithiocarbamates [17,18], triazoles [19], saccharinates [11,15], terpyridines [20], benzothiazoles [21,22], Schiff bases [23], benzimidazoles [24], imides [25,26,27,28] and others [10,29], as well as metal ions such as palladium, ruthenium, nickel, rhodium, iridium, gold and copper, have all been used in the development of new coordination compounds or metal complexes by researchers taking a biological approach [5,30,31,32]. At the same time, functionalization processes have also been developed for the controlled administration of drugs from existing complexes; these include liposome encapsulation [33], macrocycles, metal nanoparticles and carbo nanotubes [34,35], as well as bioconjugation [35].

The design of novel complexes with similar or better antitumor activity than that of platinum(II) drugs but with reduced side effects can be seen as an ongoing challenge. With this in mind, the use of biometals such as copper(II) to prepare new complexes as drugs against cancer is now a hot topic around the world. Copper is now considered an essential biometallic element; it is found in most aerobic organisms, where it carries out important biological functions as a structural and catalytic cofactor [36,37,38].

**Figure 2:**
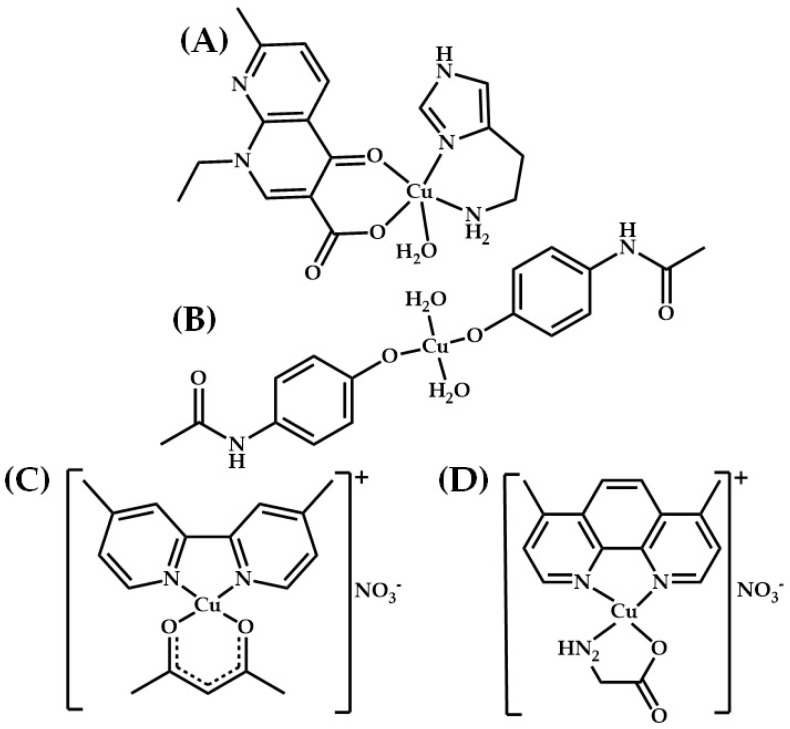
Structures of some copper(II)-based complexes with biological activity: antimicrobial (**A**), anti-inflammatory (**B**) and antiproliferative or antitumoral (**C**,**D**).

Copper(II)-based complexes, such as those shown in Figure 2, have been reported to have important biological activities which may be antimicrobial (Figure 2A) [39], anti-inflammatory (Figure 2B) [40], antiproliferative or antitumoral (Figure 2C,D) [30]. These types of complexes are therefore key objects of study in the field of bioinorganic science. To the best of our knowledge, there were almost no reports on the use of copper(II)-based complexes in clinical trials prior to 2014 [41]. Casiopeinas^®^ is a family of more than 100 copper(II) compounds designed by Mexican scientists [42]. Some compounds in this group have demonstrated antiproliferative and antineoplastic activity both in vivo and in vitro. Such activities are due to genotoxic and cytotoxic effects mediated by mechanisms such as interaction with DNA, action nucleases, production of ROS, and mitochondrial imbalance. However, Casiopeinas^®^ compounds do not produce significant cytotoxic effects against normal cells [24,43]. There is great interest in this group of compounds due to their potential as antitumor agents. In recently reported preclinical studies for CasIII-Ia and CasII-gly, these compounds showed promising results, opening the way to their being selected for phase I clinical trials in Mexico [44,45,46].

The challenge of discovering new antineoplastic metallodrugs which are more effective than those based on platinum(II) is ongoing. Today, many research groups are engaged in the quest for such drugs, in the ultimate hope that the health of humans, animals and even plants might be enhanced. In this review, we present a meta-analysis of research on Pt(II)- and Cu(II)-based complexes published in academic papers between 2014 and 2023. Brief descriptions of their biological activities and possible targets of action are also included.

## 2. General Results of the Meta-Analysis

**Figure 3:**
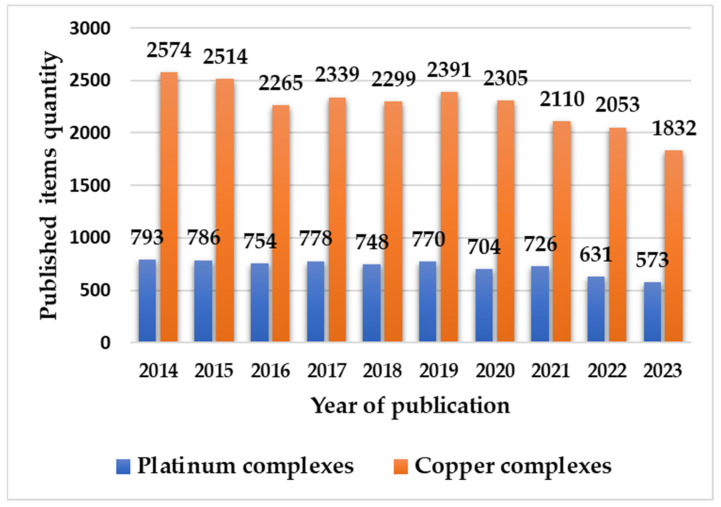
Results of meta-analysis of papers published between 2014 and 2023 on platinum- and copper-based complexes/compounds, as found on the Web of Science search engine.

Many coordination compounds containing transition metals such as Pt(II) and Cu(II) have been described in the literature, and these have shown a wide range of biological activity with specific target cells, including biomolecules and other cellular components. Figure 3 presents overall results of the meta-analysis conducted for the present study. Our search was carried out with the Web of Science search engine, using the following combinations of words: “Complexes platinum II OR coordination compounds platinum II AND biological activity” and “Complexes copper II OR coordination compounds copper II AND biological activity”. The search covered the ten years from 2014 to 2023 and revealed 7263 published papers about platinum(II)-based complexes, in addition to 22,682 such papers concerning copper(II)-based complexes, as indicated by the blue and orange bars, respectively, in Figure 3.

The results showed that three times as many papers were published on copper(II) complexes than platinum(II) complexes. Several factors may be considered when interpreting this finding. In economic terms, copper(II) reagents generally have a lower cost than platinum(II) reagents [47]. In addition, the higher toxicity of platinum(II), compared with copper(II), results in more costly laboratory security arrangements [47]. In terms of bioinorganic chemistry, Cu(II) can be seen as an essential micronutrient within biological systems, where it plays an important role as part of some metalloenzymes, making it a possible substitute for platinum(II) [11,12]. Another factor concerns the different chemical mechanisms required to prepare the copper(II) and platinum(II) complexes [11,13,19]. In addition, it can be seen from Figure 3 that there was a downward trend in published papers on both metals between 2019 and 2023; this can be attributed to the effects of the COVID-19 pandemic. Despite this, the design of complexes based on platinum(II) or copper(II) continues to be a hot research topic, as evidenced by the fact that 22 papers on platinum(II) and 90 papers on copper(II) have already been published in 2024.

A major goal of this review was to share important findings in published papers identified in the meta-analysis described above. Specifically, we sought to highlight recent work on platinum(II)- and copper(II)-based complexes in which important biological activities and specific target sites were considered. We therefore present in Table 1 a list of platinum(II) and copper(II) complexes described in recent studies, along with targets of action which will be further considered in the following sections of this paper. Corresponding chemical structures for the platinum(II) and copper(II) compounds are provided in Table S1 of the Supplementary Materials.

## 3. Mechanisms of Action of Platinum(II) and Copper(II) Complexes

### 3.1. Platinum(II)

Many platinum(II)-based complexes have been described and evaluated in clinical studies in recent years. It may be suggested that researchers have maintained a high level of interest in these complexes because they are now used for the treatment of most types of cancer [5], despite their known side effects [1,10]. This situation has motivated the design of new metallodrugs, involving different ligands and metals, so that chemical structures might be obtained which can interact with specific biological targets.

There are different theories about how cisplatin is able to kill cells; however, deoxyribonucleic acid (DNA) is known to be the main target of platinum(II)-based drugs with anticancer activity. The cisplatin interacts with the DNA double helix and acts upon cytotoxic lesions through the formation of interstrand cross-linking-type DNA-cisplatin adducts. These adducts are formed by the binding to nitrogen of guanine and adenine nucleotides. First, the “aquation or hydroaquation” process of cisplatin takes place inside the cell. In this process, the two chloride ions of cisplatin are displaced by the water molecules, forming an aquo-complex. The water molecules are then easily displaced by other nucleophilic functional groups, including some of the aforementioned nucleotides. On the other hand, the trans isomer of cisplatin is incapable of forming this type of adduct; consequently, it does not exhibit effective antitumor activity [75].

**Figure 4:**
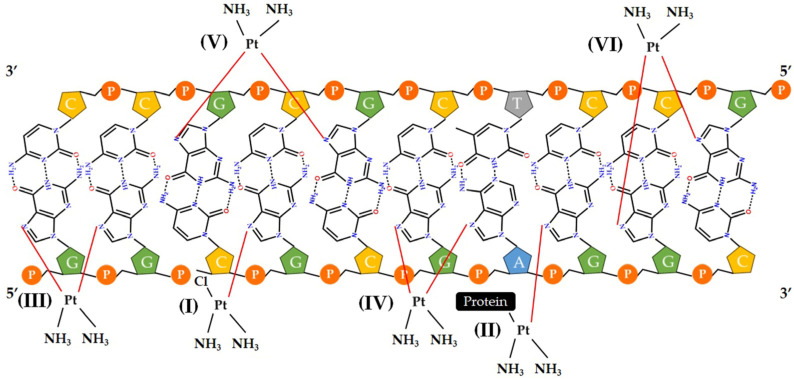
Adducts formed by different types of binding between DNA (phosphate groups: orange circles; nucleosides adenosine, guanosine, thymidine and cytidine: blue, green, gray and yellow pentagons) and cisplatin, where the monofunctional and bifunctional binding modes correspond to (I), (II) and (III), (IV), (V), respectively.

Six different types of binding (I to VI) between DNA and cisplatin have been reported, as illustrated in Figure 4.

In the I and II types, platinum(II) binds to only one nucleotide at one site (monofunctional); in the III, IV, V and VI types, Pt(II) binds to two nucleotides at two sites; these may be the same or different, and they can be on the same or a different DNA strand (bifunctional) [5,75,76]. Furthermore, platinum(II)-based complexes can interact with DNA in different modes, including non-covalent interactions, depending on the design of the chemical structure and the type of ligands selected. These forms of action are described in Section 3.3.

### 3.2. Copper(II)

Since ancient times, copper(II) salts have been used in the treatment of various fungal, viral and bacterial infections [77]. Due to the biological activity of Cu(II), several complexes based on this metal have been designed. In general, with regard to copper(II)-based complexes, there have been only a few reports on ongoing clinical trials of their use as metallodrugs. However, the situation is different for complexes containing ligands. These have been extensively tested in phase I, II and III clinical trials for the treatment of various diseases such as Alzheimer’s and Menkes disease, and their effectiveness as antineoplastics has also been researched [78,79,80,81,82,83]. The most representative copper(II) complexes with antitumor potential are CasIII-Ia and CasII-gly from the family of Casiopeines^®^ (Figure 2C,D); these are now in phase I clinical trials in Mexico [30].

**Figure 5:**
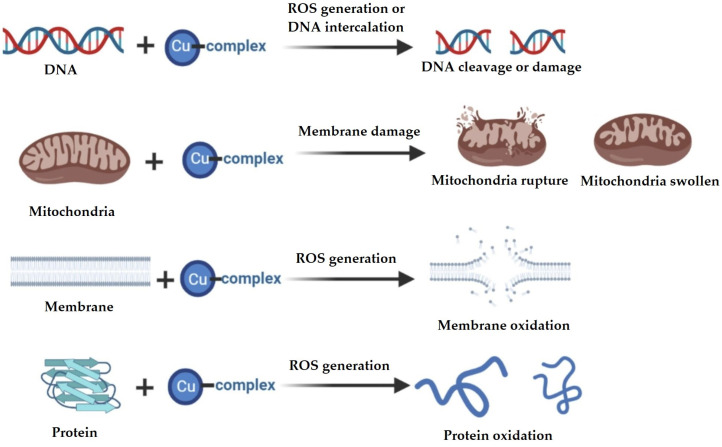
Biological targets of copper(II)-based complexes [11].

For copper (II)-based complexes, different biological targets have been described; these include cellular components such as DNA, mitochondria, cell membranes and certain proteins, as illustrated in Figure 5.

The biological targets selected by the copper(II) complexes depend on their chemical structures and the geometry around the metal centers, as well as the ligands involved. It is well known that the mechanisms of action of copper(II)-based complexes are mediated by the generation of reactive oxygen species (ROS) resulting from the oxidation–reduction capacity of copper, i.e., its ability to change from Cu(II) to Cu(I) [11,36].

As with platinum(II)-based complexes, copper(II)-based complexes can also interact with DNA in different modes, including non-covalent interactions, depending on the design of the chemical structure and the type of ligands selected. These forms of action may now be described.

### 3.3. DNA Binding Modes of Metal-Based Complexes

**Figure 6:**
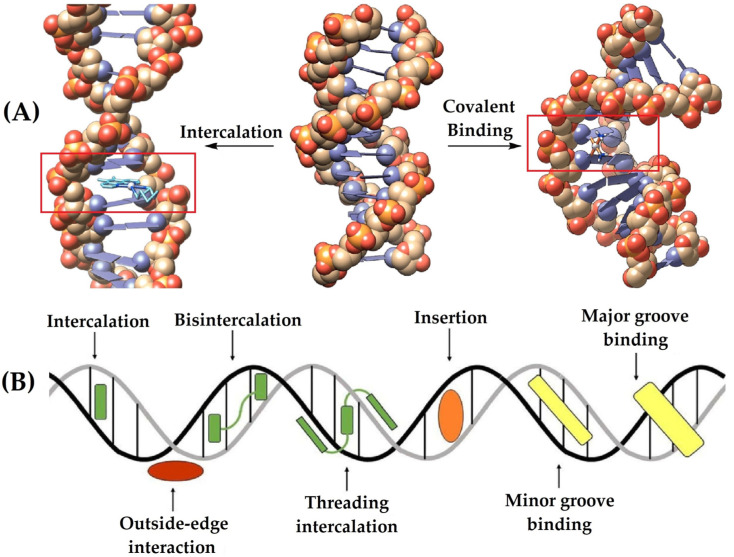
Modes of interaction between metal-based complexes and the DNA structure: in a covalent or non-covalent manner (**A**), and different types of the non-covalent form (**B**) [76,85].

As stated above, metal-based complexes can interact with the DNA structure in a covalent or non-covalent manner, as shown in Figure 6A. The covalent form occurs when there is a direct union between the metal and specific parts of the DNA structure, such as nucleotides, riboses or phosphate chains, as has been reported for the mechanism used by cisplatin [5]. In the case of the non-covalent form, there are different types of interactions which are mainly mediated by the ligands present in the metal-based complexes. These ligands can interact via hydrogen bonds or other intramolecular forces; they give rise to intercalation-type interactions, bindings with major and minor grooves, and insertion or electrostatic unions, as shown in Figure 6B [76,84,85].

## 4. Recent Studies on Platinum(II) and Copper(II) Complexes and Their Target Sites

### 4.1. Platinum(II)

#### 4.1.1. Target Site: DNA

The meta-analysis conducted for the present study produced 1647 results relating to platinum(II) complexes and their DNA interactions. Some of these are mentioned in Table 1. Our review revealed that platinum(II) complexes show promising biological activity as anticancer agents, with mechanisms of action that involve the double-helix structure of DNA. Reported complexes mainly showed in vitro activity against various cancer cell lines. Their activity was attributed to a wide variety of covalent and non-covalent interaction modes presented by the complexes. Researchers have synthesized monofunctional platinum(II)-based complexes and evaluated their biological activity. Such complexes form covalent adducts with the DNA structure, and undergo aquation inside the cell, causing displacement of a chloride ion ligand by a water molecule ligand; this enables union with nitrogenous bases, specifically the nitrogen 7 of guanine [22,48,49,50,51]. In other studies, complexes have been identified which act as intercalating agents because they have the capacity to be introduced between nitrogenous bases of DNA. This intercalation mechanism has mainly been reported for complexes with planar ligands and aromatic systems [14,18]. Furthermore, complexes that interact with DNA through the major and minor grooves have also been reported [54]. Both types of non-covalent interactions (intercalation and groove interaction) are mediated by hydrogen bonds or other intramolecular forces. The majority of reported examples have been mononuclear complexes, but some binuclear complexes have also been identified [14,23]. These interact only weakly with the DNA structure on account of their size, and their cytotoxic activity is attributed to other pathways, such as the inhibition of proteins [23].

#### 4.1.2. Target Site: Mitochondria

Mitochondria are cellular organelles which are mainly responsible for providing energy to the cell. They play important roles in metabolism and in apoptosis. In cancer cells, generating a low-oxygen microenvironment limits the oxidative phosphorylation process in energy generation, forcing cells to increase the glycolysis process (aerobic process) to compensate for the energy deficiency (Warburg effect). Because this is found only in cancer cells [86], it has become a key area of study in the design of new anticancer drugs. Platinum(II)-based complexes that have mitochondria as their target site have been explored with the objective of interrupting the aforementioned functions. Researchers have found that these kind of complexes contain ligands such as phosphines in their structure that have a positive charge [48,49,50,52,56]. These complexes have been shown to exhibit biological activity and accumulation in mitochondria. It has been suggested that the maintained negative microenvironment of the mitochondria facilitates the biological activity of the complexes and allows their entrance into the organelles [86]. Furthermore, complexes with ligands such as saccharinate, the naphthyl group [17,20], benzopyran and benzothiazole [15,16], as well as mixed complexes with ligands such as lonidamine [53] and tacrine [57] have also been reported. In general, we may say that the above-mentioned platinum(II)-based complexes which have mitochondria as a target site are mediated by mechanisms such as membrane depolarization, loss of ultrastructure, and membrane dysfunction, as well as the generation of ROS that induces apoptosis.

#### 4.1.3. Target Site: Proteins and Enzymes

Our review also revealed reports of platinum (II)-based complexes with other sites of action, including protein residues and enzymes, which are known to play important roles in different processes of cellular metabolism. To date, these have not been the main target sites in the design of platinum(II)-based anticancer drugs; however, they have been used as strategic and specific target sites. Complexes that exhibit biological activity against various cancer cell lines have been reported. It has been suggested that such cytotoxic activity is due to mechanisms that inhibit enzymes, such as topoisomerase [14,50] and telomerase [57], which cause damage and prevent DNA repair. Such complexes may also bind to proteins in active sites such as phosphatases, inhibiting their activity and causing DNA damage [23], and they may also bind to proteins such as human serum albumin [21].

### 4.2. Copper(II)

#### 4.2.1. Target Site: DNA

Our literature review revealed 4130 papers that considered copper(II) complexes and their interactions with DNA. Researchers have highlighted a number of copper(II)-based complexes which show promising biological activity as anticancer agents. The cytotoxic activities exhibited by different complexes are attributed to different modes of action that depend on their chemical structures and the ligands present in the complex. Binuclear copper(II) complexes have been shown to have the ability to bind to the phosphate chain of DNA through two adjacent sites [58], or to function as intercalating agents between nitrogenous bases [30,61,72], depending on the type of ligand present. The activity of mononuclear copper (II) complexes has been attributed to intercalatory interactions with DNA resulting from the planar aromatic nature of the ligands [65,67], as well as the interaction through the minor groove of DNA mediated by some types of ligands [30,67,72]. Such interactions serve to inhibit the processes of DNA replication and repair. Researchers have also described copper(II) complexes that act through other intracellular pathways, such as the generation of ROS, indirectly causing DNA damage [61,65,70,71,72]. All these forms of copper(II) complexes act directly on the cell to activate programmed cell-death pathways.

#### 4.2.2. Target Site: Mitochondria

It has been reported that copper(II)-based complexes that have the mitochondria as their target site play important roles in the aforementioned cellular processes. These complexes act mainly through mechanisms such as the generation of ROS mediated by the oxidation–reduction capacity of the copper ion (Cu^2+^, Cu^1+^) [30,59,60,72], depolarization of the mitochondrial membrane [60,61], changes in the permeability of the mitochondrial membrane that activate the mechanism of mitophagy (degradation and selective recycling of mitochondria) [68,69], and generalized mitochondrial dysfunction [70,71], as well as the release of apoptotic factors [62] that lead the cell to apoptosis. These processes are mainly induced by the accumulation of copper(II)-based complexes within the mitochondrial matrix.

#### 4.2.3. Target Site: Proteins and Enzymes

Our review also revealed reports of copper(II)-based complexes with other sites of action, including protein residues and enzymes. To date, these have not been the main target sites in anticancer drug design; however, they have been used as strategic and specific target sites. Researchers have identified copper(II)-based complexes that inhibit enzymes such as the topoisomerases responsible for the topological control of DNA during the replication and transcription processes [63,64,66,74], the telomerases responsible for the telomere lengthening [70] and the cyclooxygenase-2 enzyme that plays an important role in the inflammatory process [71]. These enzymes are of interest because they are highly active in cancer cells. These enzymes are also of biological importance, along with ribosyltransferase (3GEY) and EGFR tyrosine kinase (1m17), due to their participation as a model in antimicrobial and anticancer studies [73]. In addition, compounds that bind to model transporter proteins such as bovine serum albumin (BSA) [63,64] and human serum albumin [67] have been reported, providing information on their behavior and transport within the human body.

## 5. Conclusions

In the present study, we reviewed recent published research on the biological activity and action targets of Pt(II) and Cu(II) complexes. This is an important area of contemporary study because almost 50% of cancer patients receiving chemotherapy are now treated with platinum(II)-based complexes. However, patients whose lives are saved by such means may experience side effects caused by the metallodrugs used. In some cases, patients prefer to avoid chemotherapy in favor of treatment with alternative non-prescribed substances. Regarding the limitations associated with the antineoplastic and antimicrobial metallodrugs which are currently used, different paths may be taken to overcome them. One strategy has been called “functionalization of the complexes”. This has been used to improve the characteristics of pharmaceutical drugs, for example, by better facilitating their distribution and delivery within biological systems. In addition, complexes are now being redesigned by selecting new ligands and different metal ions in their structures, to improve their specificities and cytotoxicity for certain tissues, cells, or organelles.

The meta-analysis described in this paper showed that this is a hot topic of scientific interest around the world, because we found abundant papers on both metals, with a ratio of three to one between copper(II) and platinum(II), respectively. This can be attributed to several factors. From an economic point of view, it may be noted that, in 2024, platinum(II) salts are approximately 400 times more expensive per gram than copper(II) salts. Furthermore, the biological activity of platinum(II) has been more widely studied, which is why it is used in the design of anticancer metallodrugs, and its use is more refined and specific. In this review, we identified the main action targets of Pt(II) and Cu(II) metallodrugs with cytotoxic activity mentioned in recent studies. DNA seems to be the most important of these, followed by mitochondria and, finally, enzymes and proteins. Platinum(II)-based complexes have DNA as their main target site, where they interact through covalent or non-covalent forms, depending on the type of ligands. These interactions promote the inhibition of replication and transcription processes in the cell. However, we also found that copper(II)-based complexes may be considered multi-targeting, because they act simultaneously on several cellular components such as DNA, mitochondria, and proteins and enzymes, as well as promoting the generation of ROS with the aim of generating a specific cytotoxic inhabitant.

In conclusion, we may say that there has been much progress in the design of new coordination compounds with potential as antineoplastic and antimicrobial agents, based on the individual properties of each metal and ligand, with the aim of obtaining complexes with greater effectiveness and specificity. With respect to target sites, future designs of desired coordination compounds or metal complexes must consider a careful selection of ligands and metals that will allow the right interactions, so that the effects of the biological activities of metallodrugs may be better employed to cure illnesses such as cancer.

## Figures and Tables

**Table 1:** List of selected Pt(II) and Cu(II) complexes and their target sites, as identified in the conducted meta-analysis.

Formula	Targets of Action	Reference
Platinum(II)		
C_23_H_26_ClNO_3_P_2_PtS	DNA/Mitochondria	[48]
C_25_H_21_ClNO_2_PPt·H_2_O	DNA/Mitochondria	[49]
(C_24_H_27_ClN_3_PPt)(NO_3_)_2_	DNA/Mitochondria/Topoisomerase	[50]
C_44_H_41_Cl_4_N_16_O_4_Pt_2_S_4_·6DMSO	DNA/PTP1B	[23]
C_26_H_25_ClN_2_O_3_Pt·H_2_O	Mitochondria	[17,20]
C_22_H_14_Cl_2_N_4_Pt	DNA	[18]
C_18_H_17_ClN_4_OPt	HSA	[21]
(C_46_H_36_N_6_O_2_Pt_2_I_2_)^2+^	DNA/Topoisomerase	[14]
C_31_H_33_ClN_3_PPtS_2_	DNA	[22]
C_29_H_25_ClF_3_N_2_OPPtS_2_	DNA	[51]
C_14_H_9_Cl_3_N_2_OPt	Mitochondria	[15]
C_40_H_31_ClN_3_PPt	Mitochondria	[52]
C_16_H_9_Cl_2_FN_2_OPtS	Mitochondria	[16]
C_45_H_37_Cl_3_N_7_OPt·NO_3_	Mitochondria	[53]
C_50_H_50_N_2_O_6_P_2_PtS_2_	DNA/Mitochondria	[54]
C_53_H_55_N_3_O_4_PPt·ClO_4_	Mitochondria	[55,56]
C_42_H_29_N_6_Pt·Cl	Mitochondria/Telomerase	[57]
Copper(II)		
C_44_H_46_N_6_Cu_2_O_6_·9.75H_2_O	DNA	[58]
C_53_H_49_BCuF_2_I_2_N_5_O_6_·Cl	Mitochondria	[59]
C_26_H_26_Br_3_Cu_3_N_8_S_2_	Mitochondria	[60]
(C_17_H_18_BrCuN_5_S)_2_	AND/Mitochondria	[61]
C_29_H_24_Cu_2_F_2_N_6_O_11_	Mitochondria	[62]
C_15_H_12_BrClCuN_4_·DMF	Topoisomerase/BSA	[63,64]
C_54_H_42_CuN_6_O_2_·(NO_2_)_2_	DNA	[65]
Cu(C_14_H_8_Cl_2_F_3_N_2_S)_2_·3H_2_O·0.5DMF	Topoisomerase	[66]
C_26_H_17_BrClCuN_5_O_1_S·C_3_H_6_O	DNA/HSA	[67]
(C_40_H_31_Br_2_CuN_3_P)Br	Mitochondria	[68,69]
C_30_H_16_Cl_2_CuN_4_O_4_	DNA/Mitochondria/Telomerases	[70]
C_39_H_39_CuN_3_O_9_	DNA/Mitochondria/COX-2	[71]
C_17_H_19_CuN_2_O_2_·H_2_O·NO_3_	DNA	[72]
C_16_H_16_CuN_3_O_2_·2H_2_O·NO_3_	DNA	[72]
C_30_H_29_Cl_2_CuN_8_O_2_S_2_	3GEY/1m17	[73]
C_32_H_36_N_8_O_10_S_2_Cl_2_Cu	Topoisomerase	[74]

PTP1B—protein tyrosine phosphatases 1B; HSA—human serum albumin; BSA—bovine serum albumin; COX-2—ciclooxiygenase-2; 3GEY—ribosyltransferase and 1m17.

## Supplementary Materials

The following supporting information can be downloaded at: https://www.mdpi.com/article/10.3390/molecules29051066/s1, Table S1: List of Pt(II) and Cu(II) complexes found and their molecular structure. References [14,15,16,17,18,20,21,22,23,48,49,50,51,52,53,54,55,56,57,58,59,60,61,62,63,64,65,66,67,68,69,70,71,72,73,74] are cited in the Supplementary Materials.
