# Supplementary material for: Recently Reported Biological Activities and Action Targets of Pt(II)- and Cu(II)-Based Complexes
Source: Molecules. 2024 Feb 29;29(5):1066. doi: 10.3390/molecules29051066 (PMC10934961; doi:10.3390/molecules29051066)
Supplement: Supplementary file 1 [file molecules-29-01066-s001.zip › molecules-2875487-supplementary.pdf]

molecules

MDPI

Supplementary Materials

Biological Activity and Action Targets of Recent Complexes of Pt(II) and Cu(II)

Cristhian Eduardo Maciel-Flores[1], Juan Antonio Lozano-Alvarez[2] and Egla Yareth Bivián-Castro[1]*

1. Universidad de Guadalajara, Centro Universitario de los Lagos, Av. Enrique Díaz de León 1144, Col. Paseos de la Montaña, 47460, Lagos de Moreno, Jalisco, México
2. Departamento de Ingeniería Bioquímica, Universidad Autónoma de Aguascalientes. Av. Universidad 940 Cd. Universitaria, Aguascalientes 20131, Aguascalientes México

* Correspondence: egla.bivian@academicos.udg.mx; Tel.: +52-(474)-742 4314

Table S1. List of Pt(II) and Cu(II) complexes found and their molecular structure.

Formula	Structure	Ref
$C_{23}H_{26}ClNO_3P_2PtS$		[1]
$C_{25}H_{21}ClNO_2PPt \bullet H_2O$		[2]
$(C_{24}H_{27}ClN_3PPt)(NO_3)_2$		[3]
$C_{44}H_{41}Cl_4N_{16}O_4Pt_2S_4 \bullet 6DMSO$		[4]

$C_{26}H_{25}ClN_2O_3Pt{\bullet}H_2O$	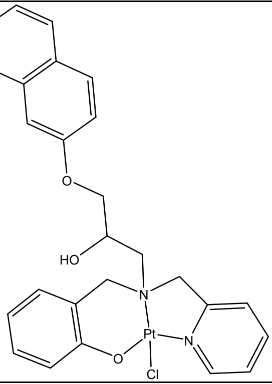	[5,6]
$C_{22}H_{14}Cl_2N_4Pt$	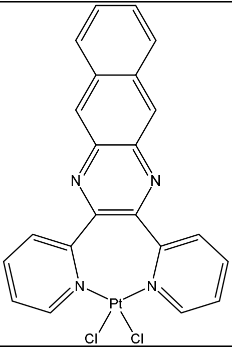	[7]
$C_{18}H_{17}ClN_4OPt$	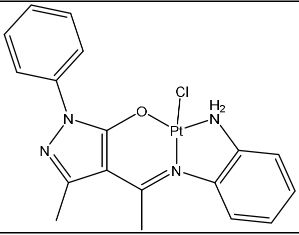	[8]
$(C_{46}H_{36}N_6O_2Pt_2I_2)^{2+}$	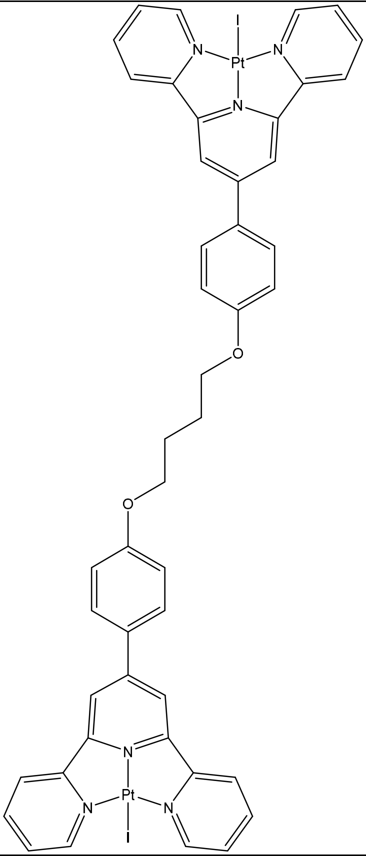	[9]

$C_{31}H_{33}ClN_3PPtS_2$	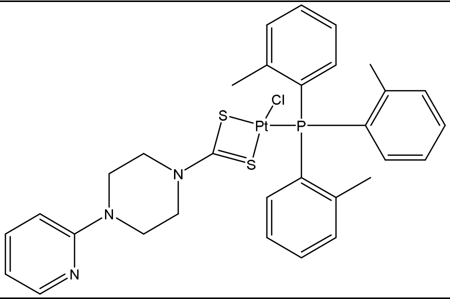	[10]
$C_{29}H_{25}ClF_3N_2OPPtS_2$	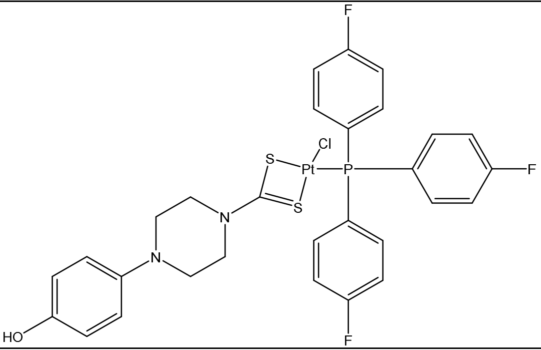	[11]
$C_{14}H_9Cl_3N_2OPt$	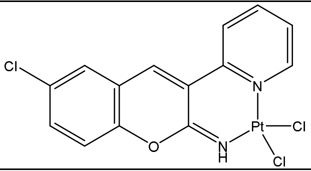	[12]
$C_{40}H_{31}ClN_3PPt$	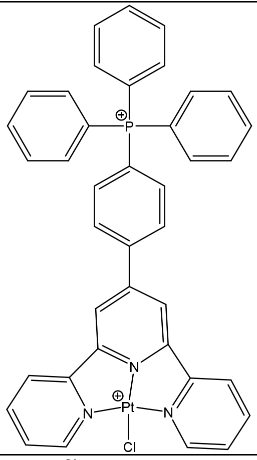	[13]
$C_{16}H_9Cl_2FN_2OPtS$	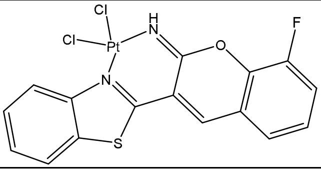	[14]

$C_{45}H_{37}Cl_3N_7OPt \bullet NO_3$	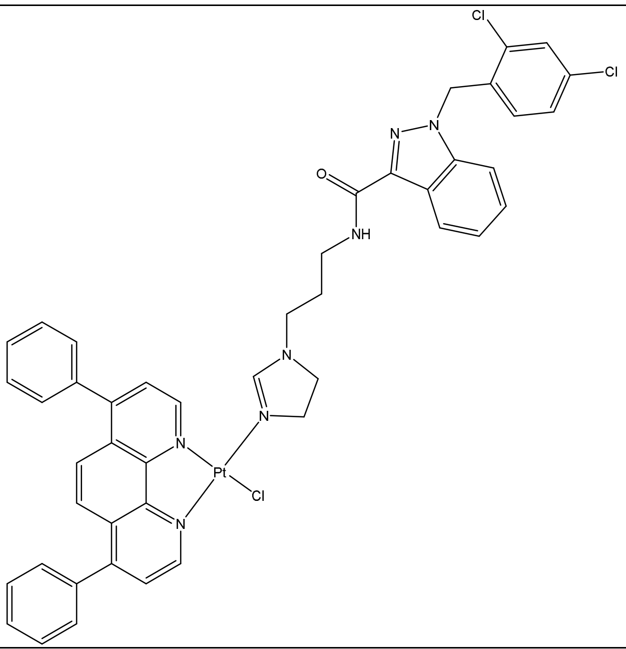	[15]
$C_{50}H_{50}N_2O_6P_2PtS_2$	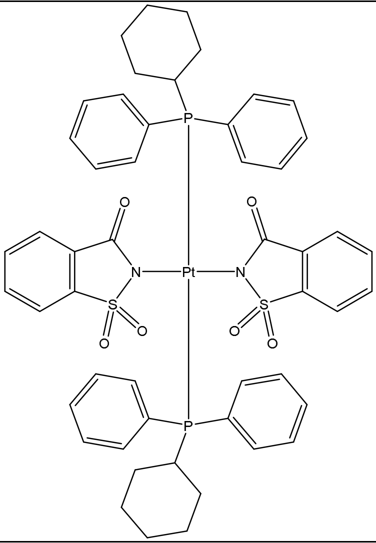	[16]
$C_{53}H_{55}N_3O_4PPt \bullet ClO_4$	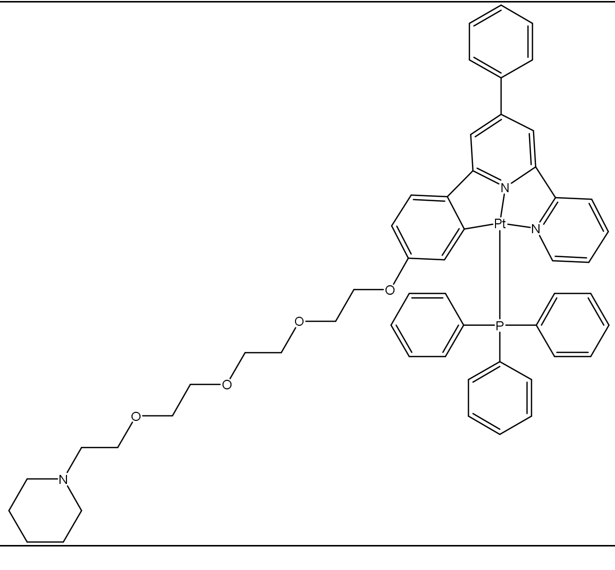	[17,18]

$C_{42}H_{29}N_6Pt{\bullet}Cl$	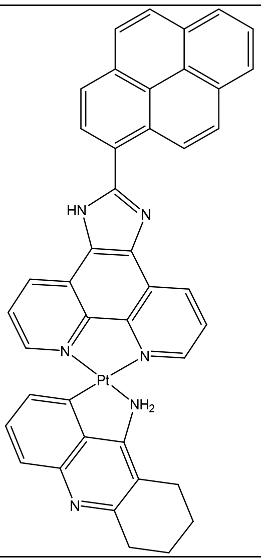	[19]
$C_{44}H_{46}N_6Cu_2O_6{\bullet}9.75H_2O$	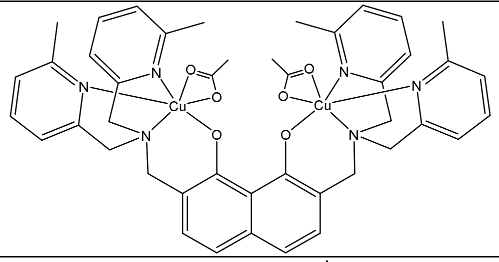	[20]
$C_{53}H_{49}BCuF_2I_2N_5O_6{\bullet}Cl$	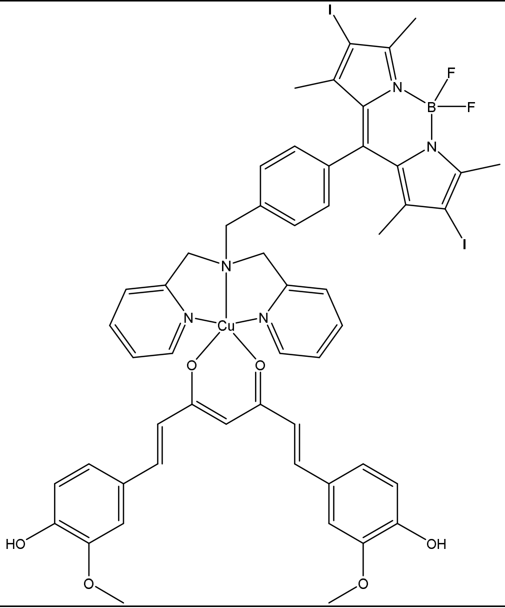	[21]
$C_{26}H_{26}Br_3Cu_3N_8S_2$	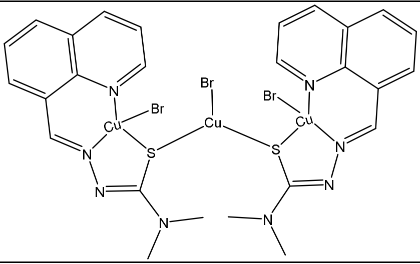	[22]

$(C_{17}H_{18}BrCuN_5S)_2$	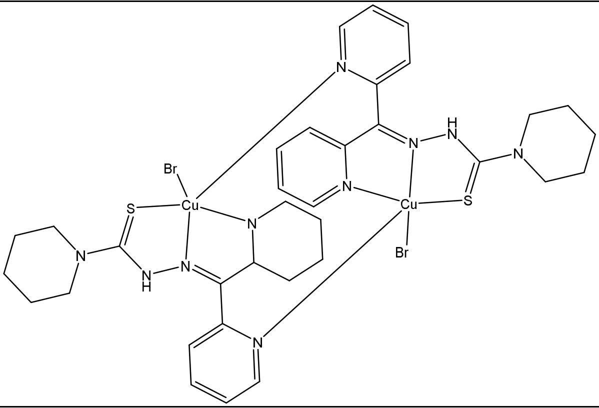	[23]
$C_{29}H_{24}Cu_2F_2N_6O_{11}$	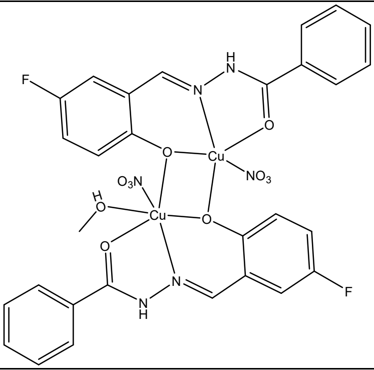	[24]
$C_{15}H_{12}BrClCuN_4$•DMF	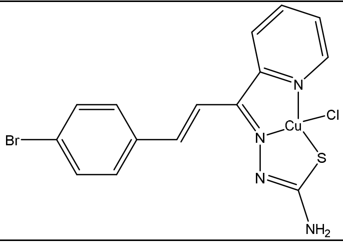	[25,26]
$C_{54}H_{42}CuN_6O_2$•$(NO_2)_2$	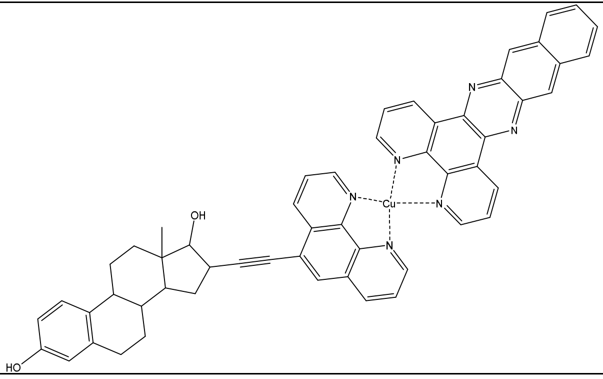	[27]

$Cu(C_{14}H_8Cl_2F_3N_2S)_2\bullet 3H_2O\bullet 0.5DMF$	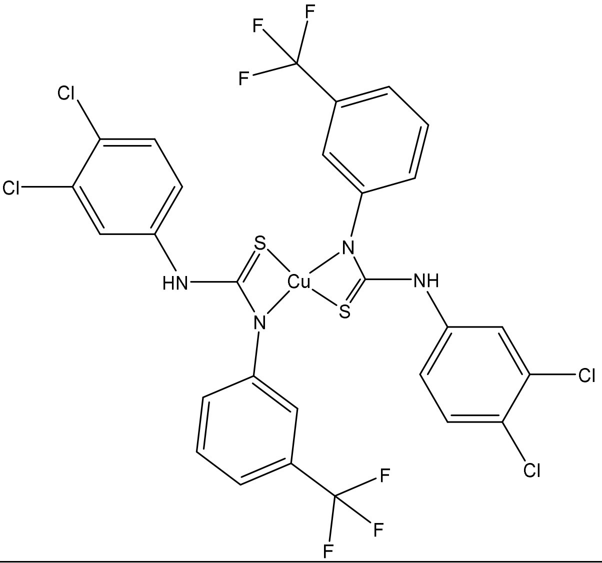	[28]
$C_{26}H_{17}BrClCuN_5O_1S\bullet C_3H_6O$	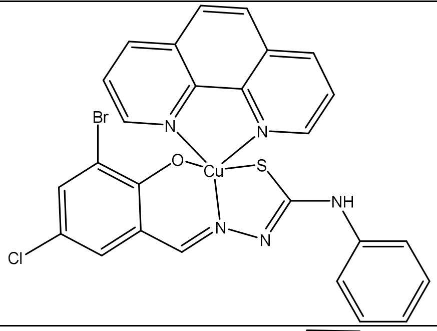	[29]
$(C_{40}H_{31}Br_2CuN_3P)Br$	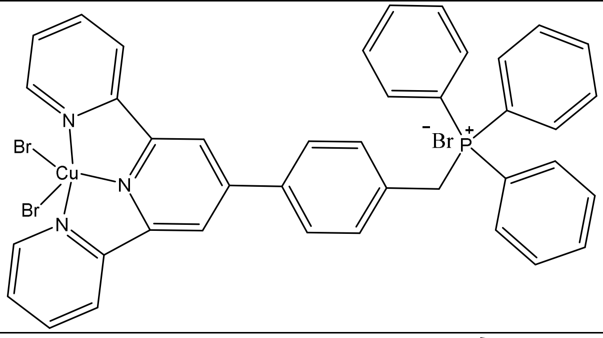	[30,31]
$C_{30}H_{16}Cl_2CuN_4O_4$	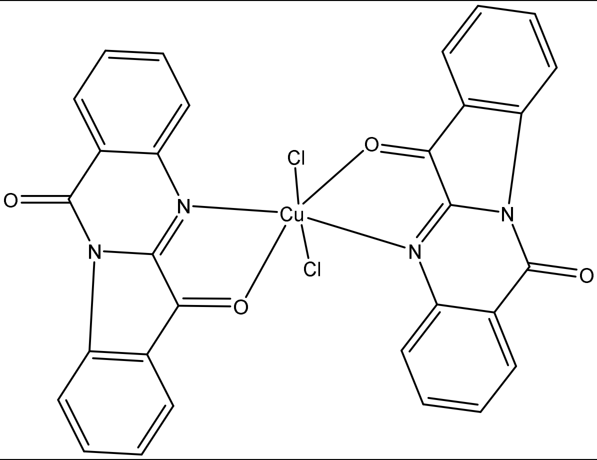	[32]

$C_{39}H_{39}CuN_3O_9$	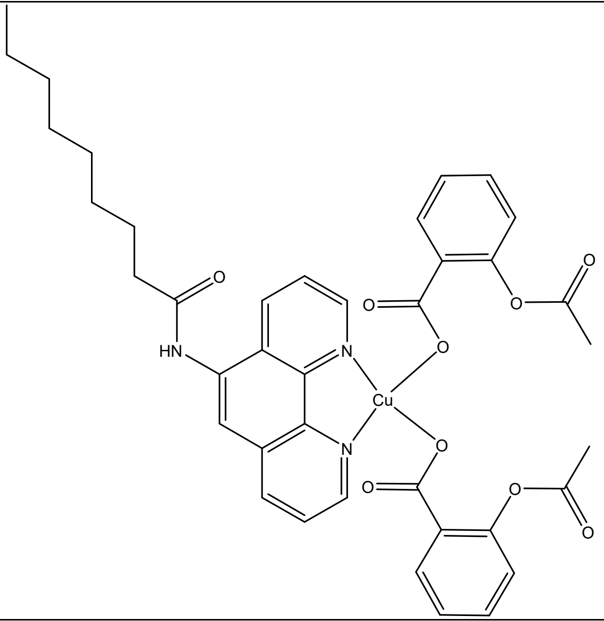	[33]
$C_{17}H_{19}CuN_2O_2{\bullet}H_2O{\bullet}NO_3$	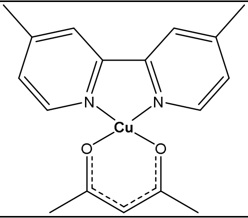	[34]
$C_{16}H_{16}CuN_3O_2{\bullet}2H_2O{\bullet}NO_3$	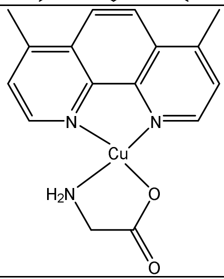	[34]
$C_{30}H_{29}Cl_2CuN_8O_2S_2$	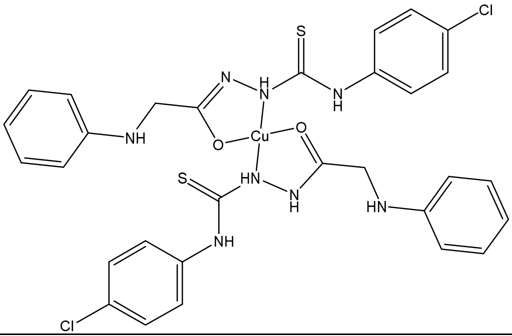	[35]

$C_{32}H_{36}N_8O_{10}S_2Cl_2Cu$	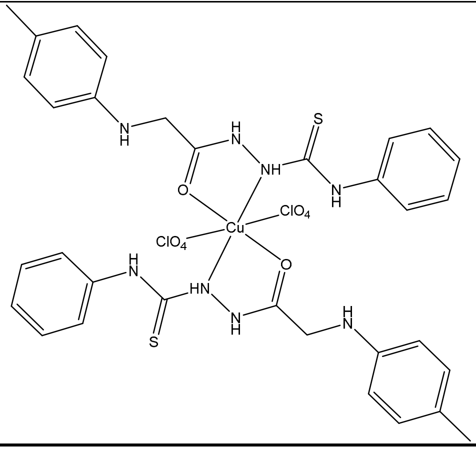	[36]

References

1. Icsel, C.; Yilmaz, V.T.; Cevatemre, B.; Aygun, M.; Ulukaya, E. Structures and Anticancer Activity of Chlorido Platinum(II) Saccharinate Complexes with Mono- and Dialkylphenylphosphines. *Journal of Inorganic Biochemistry* **2019**, *195*, 39–50, doi:10.1016/j.jinorgbio.2019.03.008.
2. Hyeraci, M.; Colalillo, M.; Labella, L.; Marchetti, F.; Samaritani, S.; Scalcon, V.; Rigobello, M.P.; Dalla Via, L. Platinum(II) Complexes Bearing Triphenylphosphine and Chelating Oximes: Antiproliferative Effect and Biological Profile in Resistant Cells. *ChemMedChem* **2020**, *15*, 1464–1472, doi:10.1002/cmdc.202000165.
3. Zhang, H.; Yang, T.; Wang, Y.; Wang, Z.; Zhu, Z.; Guo, Z.; Wang, X. DNA Topoisomerases as Additional Targets for Anticancer Monofunctional Platinum(II) Complexes. *Dalton Transactions* **2021**, doi:https://doi.org/10.1039/D0DT02608E.
4. Wang, J.; Li, X.; Yuan, C.; Su, F.; Wu, Y.-B.; Lu, L.; Zhu, M.; Xing, S.; Fu, X. Syntheses, Crystal Structures, and Biological Evaluations of New Dinuclear Platinum(II) Complexes with 1,2,4-Triazole Derivatives as Bridging Ligands. *Dalton Trans.* **2021**, *50*, 4527–4538, doi:10.1039/D0DT03285A.
5. Maciel, L.L.F.; Silva, M.B.; Moreira, R.O.; Cardoso, A.P.; Fernandes, C.; Horn, A.; De Aquino Almeida, J.C.; Kanashiro, M.M. In Vitro and In Vivo Relevant Antineoplastic Activity of Platinum(II) Complexes toward Triple-Negative MDA-MB-231 Breast Cancer Cell Line. *Pharmaceutics* **2022**, *14*, 2013, doi:10.3390/pharmaceutics14102013.
6. Moreira, R.O.; Morcelli, S.R.; Kanashiro, M.M.; Resende, J.A.L.C.; Maciel, L.L.F.; De A. Almeida, J.C.; Gahan, L.R.; Horn, A.; Fernandes, C. Modulating the Antitumoral Activity by the Design of New Platinum(II) Compounds: Synthesis, Characterization, DFT, Ultrastructure and Mechanistic Studies. *Journal of Inorganic Biochemistry* **2019**, *194*, 200–213, doi:10.1016/j.jinorgbio.2018.12.016.
7. Di Pietro, M.L.; Stagno, C.; Efferth, T.; Omer, E.A.; D'Angelo, V.; Germanò, M.P.; Cacciola, A.; De Gaetano, F.; Iraci, N.; Micale, N. Antileukemia Activity and Mechanism of Platinum(II)-Based Metal Complexes. *Molecules* **2022**, *27*, 9000, doi:10.3390/molecules27249000.
8. Abolhassan, M.R.; Divsalar, A.; Badalkhani-khamseh, F.; Kheiripour, N.; Eslami-Moghadam, M.; Mirzaei, H. Protein Binding and Anticancer Activity of Two Newly Synthesized Schiff Base Platinum (II) Complexes: A Theoretical and Experimental Study. *Journal of Molecular Structure* **2023**, *1289*, 135917, doi:10.1016/j.molstruc.2023.135917.
9. Chai, K.; Jiang, Y.; Han, T.; Niu, J.; Yao, L.; Zhang, H.; Zeng, M.; Zhang, L.; Duan, X.; Wang, J. Synthesis, DNA Binding, Topoisomerase I Inhibition and Antiproliferation Activities of Three New Binuclear Terpyridine Platinum(II) Complexes. *Polyhedron* **2019**, *157*, 124–130, doi:10.1016/j.poly.2018.09.053.
10. Imran, M.; Zia-ur-Rehman; Kondratyuk, T.; Bélanger-Gariepy, F. New Ternary Platinum(II) Dithiocarbamates: Synthesis, Characterization, Anticancer, DNA Binding and DNA Denaturing Studies. *Inorganic Chemistry Communications* **2019**, *103*, 12–20, doi:10.1016/j.inoche.2019.02.007.
11. Kashif Amir, M.; Hogarth, G.; Khan, Z.; Imran, M.; Zia-ur-Rehman. Platinum(II) Dithiocarbamate Complexes [Pt(S2CNR2)Cl(PAr3)] as Anticancer and DNA-Damaging Agents. *Inorganica Chimica Acta* **2020**, *512*, 119853, doi:10.1016/j.ica.2020.119853.
12. Liu, J.-H.; Wei, Z.-Z.; Yang, L.; Qin, Q.-P.; Liu, X.-X.; Tan, M.-X. Synthesis, Structures, and Anticancer Potentials of Four Platinum (II) Complexes with Benzopyran Derivatives Targeting Mitochondria. *Inorganic Chemistry Communications* **2020**, *122*, 108267, doi:10.1016/j.inoche.2020.108267.
13. Wang, K.; Zhu, C.; He, Y.; Zhang, Z.; Zhou, W.; Muhammad, N.; Guo, Y.; Wang, X.; Guo, Z. Restraining Cancer Cells by Dual Metabolic Inhibition with a Mitochondrion-Targeted Platinum(II) Complex. *Angew Chem Int Ed* **2019**, *58*, 4638–4643, doi:10.1002/anie.201900387.
14. Wei, Q.-M.; Wei, Z.-Z.; Zeng, J.-J.; Yang, L.; Qin, Q.-P.; Tan, M.-X.; Liang, H. Synthesis, Structures and Anticancer Potentials of Five Platinum(II) Complexes with Benzothiazole-Benzopyran Targeting Mitochondria. *Polyhedron* **2021**, *196*, 115004, doi:10.1016/j.poly.2020.115004.

15. Muhammad, N.; Tan, C.-P.; Muhammad, K.; Wang, J.; Sadia, N.; Pan, Z.-Y.; Ji, L.-N.; Mao, Z.-W. Mitochondria-Targeting Monofunctional Platinum(II)–Lonidamine Conjugates for Cancer Cell de-Energization. *Inorg. Chem. Front.* **2020**, *7*, 4010–4019, doi:10.1039/D0QI01028F.
16. Yilmaz, V.T.; Icsel, C.; Turgut, O.R.; Aygun, M.; Erkisa, M.; Turkdemir, M.H.; Ulukaya, E. Synthesis, Structures and Anticancer Potentials of Platinum(II) Saccharinate Complexes of Tertiary Phosphines with Phenyl and Cyclohexyl Groups Targeting Mitochondria and DNA. *European Journal of Medicinal Chemistry* **2018**, *155*, 609–622, doi:10.1016/j.ejmech.2018.06.035.
17. Li, J.; He, X.; Zou, Y.; Chen, D.; Yang, L.; Rao, J.; Chen, H.; Chan, M.C.W.; Li, L.; Guo, Z.; et al. Mitochondria-Targeted Platinum(II) Complexes: Dual Inhibitory Activities on Tumor Cell Proliferation and Migration/Invasion via Intracellular Trafficking of β-Catenin. *Metallomics* **2017**, *9*, 726–733, doi:10.1039/C6MT00188B.
18. Guo, Z.; Tong, W.-L.; Chan, M.C.W. Luminescent Oligo(Ethylene Glycol)-Functionalized Cyclometalated Platinum(Ii) Complexes: Cellular Characterization and Mitochondria-Specific Localization. *Chem. Commun.* **2014**, *50*, 1711, doi:10.1039/c3cc47150k.
19. Qin, Q.-P.; Wang, S.-L.; Tan, M.-X.; Wang, Z.-F.; Luo, D.-M.; Zou, B.-Q.; Liu, Y.-C.; Yao, P.-F.; Liang, H. Novel Tacrine Platinum(II) Complexes Display High Anticancer Activity via Inhibition of Telomerase Activity, Dysfunction of Mitochondria, and Activation of the P53 Signaling Pathway. *European Journal of Medicinal Chemistry* **2018**, *158*, 106–122, doi:10.1016/j.ejmech.2018.09.008.
20. Jany, T.; Moreth, A.; Gruschka, C.; Sischka, A.; Spiering, A.; Dieding, M.; Wang, Y.; Samo, S.H.; Stammler, A.; Bögge, H.; et al. Rational Design of a Cytotoxic Dinuclear Cu_2 Complex That Binds by Molecular Recognition at Two Neighboring Phosphates of the DNA Backbone. *Inorg. Chem.* **2015**, *54*, 2679–2690, doi:10.1021/ic5028465.
21. Bhattacharyya, A.; Dixit, A.; Mitra, K.; Banerjee, S.; Karande, A.A.; Chakravarty, A.R. BODIPY Appended Copper(II) Complexes of Curcumin Showing Mitochondria Targeted Remarkable Photocytotoxicity in Visible Light. *Med. Chem. Commun.* **2015**, *6*, 846–851, doi:10.1039/C4MD00425F.
22. Gu, S.; Yu, P.; Hu, J.; Liu, Y.; Li, Z.; Qian, Y.; Wang, Y.; Gou, Y.; Yang, F. Mitochondria-Localizing N-Heterocyclic Thiosemicarbazone Copper Complexes with Good Cytotoxicity and High Antimetastatic Activity. *European Journal of Medicinal Chemistry* **2019**, *164*, 654–664, doi:10.1016/j.ejmech.2019.01.014.
23. Qi, J.; Yao, Q.; Tian, L.; Wang, Y. Piperidylthiosemicarbazones Cu(II) Complexes with a High Anticancer Activity by Catalyzing Hydrogen Peroxide to Degrade DNA and Promote Apoptosis. *European Journal of Medicinal Chemistry* **2018**, *158*, 853–862, doi:10.1016/j.ejmech.2018.09.034.
24. Jiang, S.; Ni, H.; Liu, F.; Gu, S.; Yu, P.; Gou, Y. Binuclear Schiff Base Copper(II) Complexes: Syntheses, Crystal Structures, HSA Interaction and Anti-Cancer Properties. *Inorganica Chimica Acta* **2020**, *499*, 119186, doi:10.1016/j.ica.2019.119186.
25. Vutey, V.; Castelli, S.; D'Annessa, I.; Sâmia, L.B.P.; Souza-Fagundes, E.M.; Beraldo, H.; Desideri, A. Human Topoisomerase IB Is a Target of a Thiosemicarbazone Copper(II) Complex. *Archives of Biochemistry and Biophysics* **2016**, *606*, 34–40, doi:10.1016/j.abb.2016.07.009.
26. Da Silva, J.G.; Recio Despaigne, A.A.; Louro, S.R.W.; Bandeira, C.C.; Souza-Fagundes, E.M.; Beraldo, H. Cytotoxic Activity, Albumin and DNA Binding of New Copper(II) Complexes with Chalcone-Derived Thiosemicarbazones. *European Journal of Medicinal Chemistry* **2013**, *65*, 415–426, doi:10.1016/j.ejmech.2013.04.036.
27. Barrett, S.; De Franco, M.; Kellett, A.; Dempsey, E.; Marzano, C.; Erxleben, A.; Gandin, V.; Montagner, D. Anticancer Activity, DNA Binding and Cell Mechanistic Studies of Estrogen-Functionalised Cu(II) Complexes. *J Biol Inorg Chem* **2020**, *25*, 49–60, doi:10.1007/s00775-019-01732-8.
28. Bielenica, A.; Drzewiecka-Antonik, A.; Rejmak, P.; Stefańska, J.; Koliński, M.; Kmiecik, S.; Lesyng, B.; Włodarczyk, M.; Pietrzyk, P.; Struga, M. Synthesis, Structural and Antimicrobial Studies of Type II Topoisomerase-Targeted Copper(II) Complexes of 1,3-Disubstituted Thiourea Ligands. *Journal of Inorganic Biochemistry* **2018**, *182*, 61–70, doi:10.1016/j.jinorgbio.2018.01.005.
29. Shanmugaiah, M.K.; Mokkandi Palsamy, K.; Lokesh, R.; N., I.G.; Mitu, L.; Jegathalaprathaban, R.; Gurusamy, R. Ternary Copper (II) Complex Based Chemical Probes for DNA Targeting: Cytotoxic Activity under Visible Light. *Applied Organom Chemis* **2018**, *33*, e4762, doi:10.1002/aoc.4762.
30. Li, M.; Shao, J.; Guo, Z.; Jin, C.; Wang, L.; Wang, F.; Jia, Y.; Zhu, Z.; Zhang, Z.; Zhang, F.; et al. Novel Mitochondrion-targeting Copper(II) Complex Induces HK2 Malfunction and Inhibits Glycolysis via Drp1-mediating Mitophagy in HCC. *J Cellular Molecular Medi* **2020**, *24*, 3091–3107, doi:10.1111/jcmm.14971.
31. Shao, J.; Li, M.; Guo, Z.; Jin, C.; Zhang, F.; Ou, C.; Xie, Y.; Tan, S.; Wang, Z.; Zheng, S.; et al. TPP-Related Mitochondrial Targeting Copper (II) Complex Induces P53-Dependent Apoptosis in Hepatoma Cells through ROS-Mediated Activation of Drp1. *Cell Commun Signal* **2019**, *17*, 149, doi:10.1186/s12964-019-0468-6.
32. Qin, Q.-P.; Zou, B.-Q. Tryptanthrin Derivatives Copper(II) Complexes with High Antitumor Activity by Inhibiting Telomerase Activity, and Inducing Mitochondria-Mediated Apoptosis and S-Phase Arrest in BEL-7402. *New J. Chem.* **2018**, *42*, 15479, doi:https://doi.org/10.1039/C8NJ03005G.
33. Shi, X.; Fang, H.; Guo, Y.; Yuan, H.; Guo, Z.; Wang, X. Anticancer Copper Complex with Nucleus, Mitochondrion and Cyclooxygenase-2 as Multiple Targets. *Journal of Inorganic Biochemistry* **2019**, *190*, 38–44, doi:10.1016/j.jinorgbio.2018.10.003.
34. Davila-Manzanilla, S.G.; Figueroa-de-Paz, Y.; Mejia, C.; Ruiz-Azuara, L. Synergistic Effects between a Copper-Based Metal Casiopeína III-Ia and Cisplatin. *European Journal of Medicinal Chemistry* **2017**, *129*, 266–274, doi:10.1016/j.ejmech.2017.01.045.

35. Alshater, H.; Al-Sulami, A.I.; Aly, S.A.; Abdalla, E.M.; Sakr, M.A.; Hassan, S.S. Antitumor and Antibacterial Activity of Ni(II), Cu(II), Ag(I), and Hg(II) Complexes with Ligand Derived from Thiosemicarbazones: Characterization and Theoretical Studies. *Molecules* **2023**, *28*, 2590, doi:10.3390/molecules28062590.
36. Aly, S.A.; Hassan, S.S.; El-Boraey, H.A.; Eldourghamy, A.; Abdalla, E.M.; Alminderej, F.M.; Elganzory, H.H. Synthesis, Biological Activity, and the Effect of Ionization Radiation on the Spectral, XRD, and TGA Analysis of Cu(I), Cu(II), Zn(II), and Cd(II) Complexes. *Arab J Sci Eng* **2024**, *49*, 361–379, doi:10.1007/s13369-023-07988-2.

Disclaimer/Publisher's Note: The statements, opinions and data contained in all publications are solely those of the individual author(s) and contributor(s) and not of MDPI and/or the editor(s). MDPI and/or the editor(s) disclaim responsibility for any injury to people or property resulting from any ideas, methods, instructions or products referred to in the content.
